# Supplementary material for: Identification and Analysis of Micro-Exon Genes in the Rice Genome
Source: Int J Mol Sci. 2019 May 31;20(11):2685. doi: 10.3390/ijms20112685 (PMC6600660; doi:10.3390/ijms20112685)
Supplement: Supplementary file 1 [file ijms-20-02685-s001.pdf]

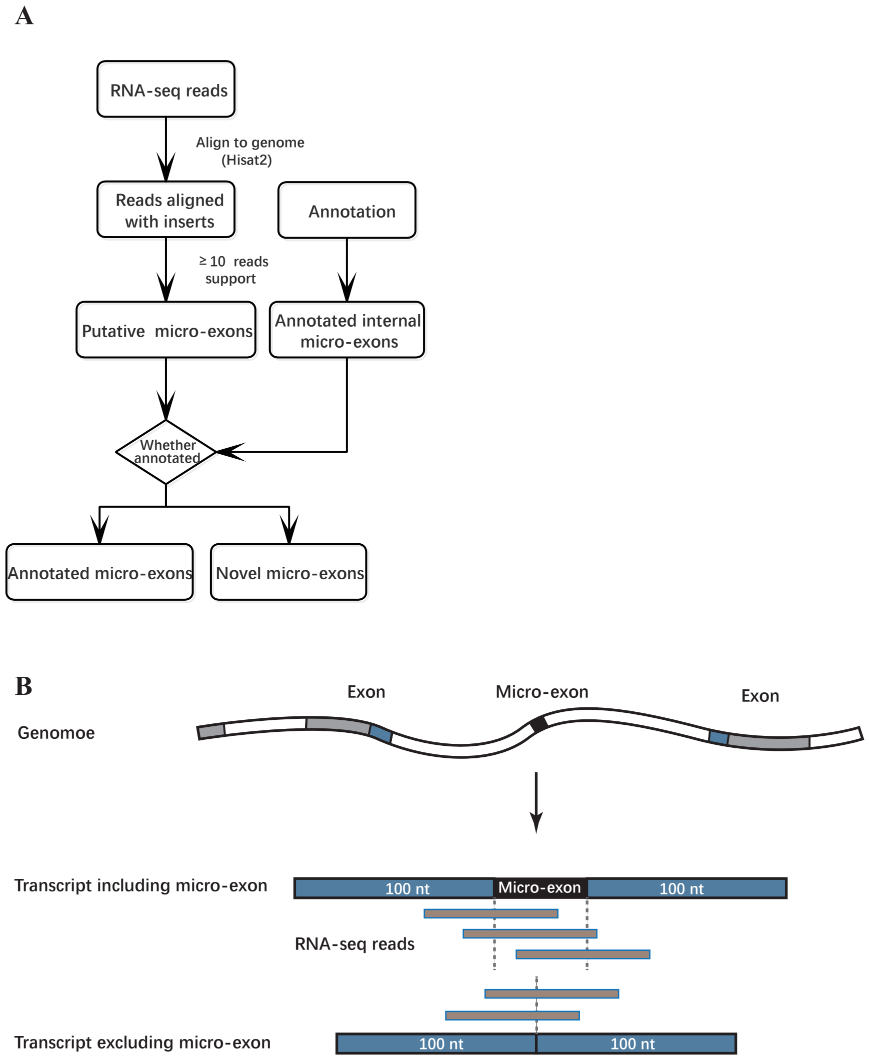

Figure S1. The pipeline of identification and PSI calculation for the micro-exons. (A) The progress of micro-exons identification. (B) The illustration of PSI calculation. Reads mapped to the left and right junctions of the micro-exon are counted as R_R and R_L, and reads mapped to the junction of the micro-exon skipping are counted as $R_{skipped}$.
